# Detection of Anxiety-Based Epileptic Seizures in EEG Signals Using Fuzzy Features and Parrot Optimization-Tuned LSTM

**DOI:** 10.3390/brainsci14080848

**Published:** 2024-08-22

**Authors:** Kamini Kamakshi Palanisamy, Arthi Rengaraj

**Affiliations:** Department of ECE, Faculty of Engineering & Technology, SRM Institute of Science and Technology, Ramapuram Campus, Ramapuram, Chennai 600089, India; kk9871@srmist.edu.in

**Keywords:** EEG epileptic seizure signals, data augmentation, fuzzy feature extraction, parrot optimization, LSTM classifier

## Abstract

In humans, epilepsy is diagnosed through electroencephalography (EEG) signals. Epileptic seizures (ESs) arise due to anxiety. The detection of anxiety-based seizures is challenging for radiologists, and there is a limited availability of anxiety-based EEG signals. Data augmentation methods are required to increase the number of novel samples. An epileptic seizure arises due to anxiety, which manifests as variations in EEG signal patterns consisting of changes in the size and shape of the signal. In this study, anxiety EEG signals were synthesized by applying data augmentation methods such as random data augmentation (RDA) to existing epileptic seizure signals from the Bonn EEG dataset. The data-augmented anxiety seizure signals were processed using three algorithms—(i) fuzzy C-means–particle swarm optimization–long short-term memory (FCM-PS-LSTM), (ii) particle swarm optimization–long short-term memory (PS-LSTM), and (iii) parrot optimization LSTM (PO-LSTM)—for the detection of anxiety ESs via EEG signals. The predicted accuracies of detecting ESs through EEG signals using the proposed algorithms—namely, (i) FCM-PS-LSTM, (ii) PS-LSTM, and (iii) PO-LSTM—were about 98%, 98.5%, and 96%, respectively.

## 1. Introduction

Epileptic seizures are recurrent [1] and can occur at any age. These seizures are analysed based on non-stationary patterns in EEG signals. Seizure prediction is a difficult task for radiologists. Signal characteristics are used for the detection of epilepsy via EEG signals. In humans, an irregular electrical signal arises due to disturbances in the brain, which cause seizures. A seizure signal’s size and amplitude vary based on the region of the brain that is affected. Epilepsy is generally classified as generalized or focal epilepsy. Genetic predispositions or brain anomalies can cause epilepsy. An epileptic person can be treated via anti-seizure drugs, lifestyle changes, and surgery. For the detection of an ES caused by anxiety, an EEG signal needs to be data-augmented.

Data augmentation [1,2,3] is used to expand a training set using generated data. Data augmentation is performed by transforming or modifying pre-existing data and thus creating new samples. With regard to EEG signals, data augmentation is used for anxiety-based EEG data creation. Random noise in an EEG signal is background noise. The channel dropout process for EEG channels is performed based on the removal of a channel. Artificial electrode artifacts are present in EEG signals, occurring in the form of abrupt spikes. For particular time intervals, seizure activity is reflected in EEG signals that are in shorter segments. These shorter segments are used for the identification of seizures within constrained temporal ranges. An EEG dataset needs to be augmented to obtain the seizure EEG data relating to anxiety disorders. Anxiety changes EEG frequency patterns. The EEG frequencies are beta, gamma, alpha, theta, and delta [4]. These frequencies change based on stress, and the types of change depend on the types of stress. Anxiety, depression, and a low mood are linked with alpha and beta signals [4]. Signs of stress can be seen in beta wave frequency changes [5]. The beta frequency range is between 12.5 and 30 Hz. Anxiety disorders increase EEG signals’ delta and theta frequencies [6]. Reductions in alpha and beta frequency levels are linked to anxiety disorders [4,6]. Anxiety is measured using the theta/beta ratio [7]. Fear, panic, anxiety, insecurity, and phobia are linked to high beta waves. Amplitude (A), width (time in seconds), mixture amplitude, and width fluctuations in EEG signals are indicative of anxiety-based seizures. The extreme learning machine wavelet auto encoder (ELM-W-AE) approach was developed by the authors of [1]. Using wavelet activation functions in conjunction with the ResNet18 classifier, it recognized and classified emotions based on EEG augmentation signals. The ELM-W-AE technique has a high classification accuracy when using wavelet functions such as the Mexican, Morlet, and Gaussian wavelets. The authors of [2] compared 13 augmented EEG sleep signals. EEG signal augmentation was performed based on the spatial domain, frequency, and time. Quantitative methods were applied for the performance evaluation of the data augmentation signals. EEG-based emotion recognition signals were obtained through data augmentation. There were a greater number of samples in the dataset, and these were fed to the Conditional Wasserstein Generative Adversarial Network (CWGAN) [3]. Epilepsy detection using DW-based filtering, feature extraction, and classification was performed using moth–flame optimization and an extreme learning machine with a multiquadric activation function. The above method had accuracies of about 92%, 95%, and 96% for three different datasets [8]. EEG signal analysis using a Maximal Overlap Discrete Wavelet Transform (MODWT), and a Multiresolution Convolutional Neural Network (CNN) yielded an 82% sensitivity and an FPR of 0.058 for the CHB-MIT dataset and an 85% sensitivity and an FPR of 0.19 for the Kaggle dataset [9]. A revised Tunable Q-Factor Wavelet Transform was applied for an EEG signal analysis in which TQWT parameters such as redundancy and Q-factor were optimized. A decision tree classifier achieved a maximum accuracy of about 99.8% when applied to the Bonn EEG dataset [10]. Xuewen Pang classified epileptic EEG signals using DWT frequency components. A support vector machine was used for EEG signal classification, achieving an accuracy of about 96.59% [11]. A fourth-order Butterworth BPF was applied to EEG signals. Filtered EEG signals were decomposed with the "db4" wavelet and denoised with a selected threshold. Feature selection was conducted using the random forest algorithm, and classification was performed using a convolutional neural network (CNN), achieving an accuracy of about 99.9% for the Bonn EEG database and about 100% for the interictal and ictal New Delhi database, respectively [12]. In [13], the data augmentation of EEG signals was used for age prediction and to increase the training dataset. The BNNSMOTE (Borderline Nearest Neighbour Synthetic Minority Oversampling Technique) [14] uses imbalanced seizure dataset signals, which consist of data augmented for convulsions and non-convulsion EEG signals, and it effectively classifies seizures. RDB-DCGAN was applied to data-augmented [15] EEG sleep signals; it improved the accuracy of sleep signal detection. Data-augmented signals improve EEG signal classification accuracy. Moreover, data augmentation solves the dataset balance problem [16]. Authors have suggested methods for classifying EEG seizure signals using neural networks and the distance between centres of gravity. The feature extraction process was performed using Daubechies’ D4 wavelet transforms. This feature selection process relies on DBCG, consisting of a fuzzy membership-weighted summation. The above technique can classify epileptic seizures in EEG signals with elevated rates of high sensitivity, accuracy, and specificity [17]. The TQWT-based decomposition of EEG signals was used for feature extraction. An autoencoder-based classification was performed using an adaptive neuro-fuzzy inference system (ANFIS) with PSO and ANFIS-BS (breeding swarm) optimization [18]. An EEG signal recognition system was created using DWT-based extracted features and the classification of normal and pathological seizures. PSO and BPNN were used for optimization and classification [19].

In [20], the authors used multilevel spectral and Multiview features in EEG seizure detection using PSO and SVM. In [21], the authors applied the tuneable-Q wavelet technique to identify and categorize EEG epileptic seizure signals. PSO and ANN were applied for the acquisition of non-linear characteristics. The accuracy was about 88.8% for TUH and 100% for the KITs database. Decision tree, random forest, and AdaBoost classifiers were used, and a hybrid optimization–control ensemble classifier was used for EEG-based seizure prediction. A hybrid optimization technique-based EEG signal feature selection had an accuracy of about 96.61% for the CHB-MIT dataset and 95.30% for the Siena scalp dataset [22]. EEG seizure signal spectral parameters were obtained through IMF and EMD. Whale Optimization Algorithm feature selection yielded an accuracy of about 97.76% using a random forest classifier [23]. Atom Search Optimization uses inertia weight and Levy Flight mechanisms during optimization. A LSSVM (least-squares support vector machine) was applied for the classification of epileptic seizures via EEG signals [24]. Seizures were categorized based on EEG signals via wavelet packet transformation, the fuzzy K-nearest neighbour fusion approach, and Hilbert transform-based modified wolf optimization. For the Bonn dataset, the accuracy was around 100%, whereas it was 99.48 ± 0.61 for the CHB-MIT dataset [25]. EEG detection using the moth–flame optimization algorithm optimized 1D convolutional neural networks (1D-CNN) for the data augmentation of EEG signals and hyperparameters tuned with MFO [26]. An EEG-based modified gorilla-troops-optimization-with-deep-learning method was used for ES prediction (MGTODL-ESP). The best feature was selected by the MGTODL-ESP model via a feature selection technique based on modified gorilla troops optimization (MGTO). A gated recurrent unit (GRU) model based on MGTO was used for the prediction of different types of ESs. The grey wolf optimizer (GWO) algorithm was used to tune the MGTODL model’s parameters [27]. A 1D CNN-LSTM approach was developed and used to identify epileptic seizures through the preprocessing and normalization of EEG signals [28]. A deep bidirectional LSTM was developed for seizure detection. Non-stationary EEG signals’ means and feature extraction were used in this method, which obtained a G-mean of about 92.66% [29]. In another study, a Hilbert vibration decomposition technique was used to extract the features from EEG epileptic seizure signals. The classification of EEG epileptic seizure signals using LSTM produced an accuracy of about 96% [30]. The authors used spectral data taken from multi-channel EEG recordings and created a two-layer LSTM for ES prediction. The 2L-LSTM model’s accuracy was about 98.14%, which is higher than that of the 1L-LSTM model [31]. The authors used a correlation-based approach for attribute selection and discrete wavelet for acquiring attributes. Applying LSTM, TUH scalp EEG data was categorized with success rates of approximately 95.08% for absence seizures and 95.92% for complex partial seizures [32]. The authors developed a time-aware CNN and recurrent neural network (TA-CNN-RNN) model for inter-ictal and ictal seizure EEG signal classification, and the LSTM attained accuracies of about 89%, 88.6%, and 88.7% for the CHB-MIT-EEG, Bonn-iEEG, and VIRGO-EEG datasets [33]. The authors used BPF and three types of feature methods, namely, Fourier transform, entropy, and approximate entropy, for the classification of epileptic and non-epileptic seizures using SVM, DT, K-NN, and NB, achieving accuracies of about 96%, 76%, 92%, and 67%, respectively [34]. In [35], the author used an RNN for the identification of epileptic seizures. The above method outperformed standard classification methods such as SVM and ANN. On the CHB-MIT and BONN datasets, the RNN-based model obtained accuracies of about 93.27% and 99.84%, respectively.

Seizures arise due to stress and anxiety, which are detected through frequency changes in EEG signals. Mid-range beta waves (15–20 Hz) are known as “beta two” waves, which are associated with increases in energy, anxiety, and performance. High beta waves (18–40 Hz) are known as “beta three” waves, which are associated with significant stress, anxiety, and high arousal [36]. An increase in the amplitude of beta wave activity is linked to anxiety and stress responses. Specifically, heightened beta activity, particularly in the right hemisphere signal regions, is correlated with anxiety disorders [36,37]. However, increased beta activity is a common finding for anxiety, and it is a potential diagnostic marker wherein higher amplitudes of beta waves correlate with greater anxiety symptoms [37,38]. Individuals with GAD (generalized anxiety disorder) often exhibit heightened beta wave activity (particularly in the beta2 signal range from 20 to 30 Hz) in the frontal and temporal regions. When augmenting EEG data, it is crucial to replicate the amplitude characteristics of real anxiety-based signals. Random data augmentation is used for synthetic data generation and maintains a realistic amplitude distribution [36]. This method minimizes the divergence between the real and generated data, ensuring the augmented signals reflect the amplitude patterns observed in authentic EEG recordings. In this paper, BONN EEG dataset signals were random data-augmented (RDA) to obtain epileptic seizure signals. Until now, researchers have not addressed the detection of seizures due to anxiety. In this study, the data augmentation of epileptic EEG signals was performed using random shifts in the signal, which mimic temporal fluctuations or misalignment in seizures due to anxiety. The random scaling of an EEG signal’s amplitude adds variances to the signal strength, suitable for varying anxiety-based seizure intensities.

The contributions of the proposed work are as follows:

1. We augmented EEG signals to obtain anxiety-based ES signals and non-epileptic signals using EEG signals from the BONN dataset. High beta waves (typically 20–30 Hz) are closely associated with anxiety, panic attacks, and heightened stress responses. Increased beta activity is often observed in individuals with anxiety disorders, indicating a state of hyperarousal and agitation [39].
2. We extracted features from data-augmented anxiety-based EEG signals using FCM and optimization algorithms, namely, (i) particle swarm optimization (PSO) and (ii) parrot optimization (PO), which were used to tune the hyperparameters of the LSTM layer and detect anxiety-related epileptic seizure signals.
3. We classified EEG ES signals and non-ES signals derived from a random augmented EEG signal dataset.

This paper is organized as follows: The EEG BONN database and techniques are discussed in Section 2, the simulation results and advantages are provided in Section 3, and a summary and conclusions are given in Section 4.

## 2. Materials and Methods

### 2.1. Dataset

**Figure 1:**
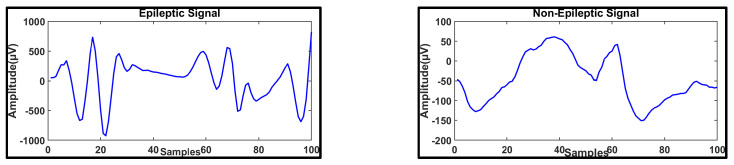
Epileptic (ES) and Non−Epileptic (non−ES) EEG signal.

**Figure 2:**
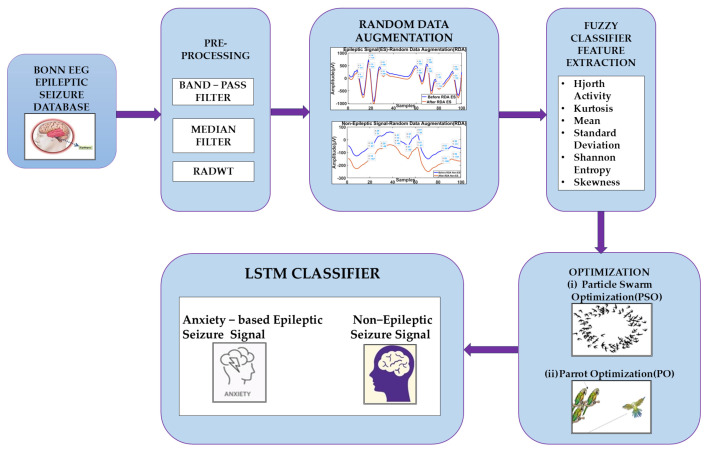
Methodology for seizure classification due to anxiety using proposed FCM−PS−LSTM methods.

In this paper, signals from the BONN EEG database [17,23,30] were data-augmented, and anxiety-related EEG seizure signals were obtained. The dataset includes 500 signals of epileptic seizures with 23.6-second durations, and all the EEG signals are classified. The BONN EEG database contains five classes of EEG signals, namely, A, B, C, D, and E, which were recorded at University Hospital Bonn (UKB), Germany. The sampling frequency of the EEG signals is about 173.61 Hz. The age and gender details of the epileptic patients are not provided in the Bonn EEG dataset. In future work, patients’ age and gender details, which are included in EEG datasets like CHB-MIT, will be considered. Table 1 depicts the BONN dataset. Figure 1 depicts both epileptic and non-epileptic EEG signals. A methodological diagram is shown in Figure 2. In the BONN dataset, data augmentation was performed on the signals in datasets E and D for anxiety-based ESs. In the proposed analysis, 70% of the EEG data were used for training, and 30% of the EEG data were used for testing.

In this study, ES and non-ES signals were filtered using a BPF (band-pass filter) and MF (median filter). RADWT was applied for the analysis of the signals. The random data augmentation technique was used to obtain anxiety-based epileptic seizure signals through arguments based on signal parameters such as amplitude (A), frequency (f), and the combination of both parameters. A fuzzy method was utilized to extract features from the augmented EEG signals. The classification was performed through the PSO-based hyperparameter tuning of LSTM for anxiety-related seizure signals. The hyperparameter tuning of LSTM classifies signals more accurately. The section below provides a detailed explanation of the methods used for the classification of anxiety-related seizure signals.

### 2.2. Pre-Processing

**Figure 3:**
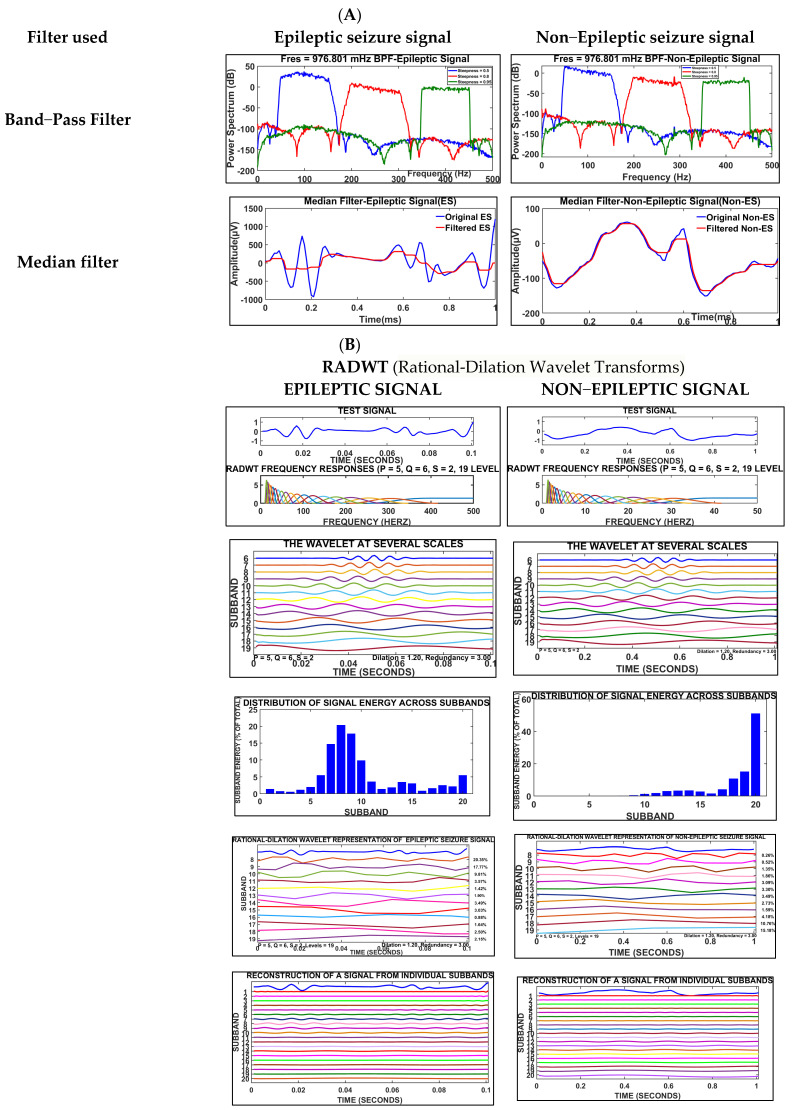
Filtered signal of EEG ES and Non−ES signal (**A**). Filtered signal (RADWT) of ES and Non−ES EEG signal (**B**).

EEG signals are pre-processed for the removal of artifacts due to blinking. Artifacts arise in frontal EEG signals due to the blinking of the eye. A band-pass filter [13,34] is used for analyses of selected frequency bands and attenuating unwanted frequencies, and abnormal frequency components in the EEG signal are filtered. This filter enhances the frequency components of EEG seizure signal activity. Signals in the passband region have a low distortion, whereas frequencies beyond the desired range are attenuated by the band-pass filter. This approach improves the accuracy of predicting EEG signals corresponding to seizures caused by anxiety. A median filter is applied using the sequence’s mean value. At a sampling frequency of 1000 Hz, artifacts are eliminated using a band-pass filter, and steepness is adjusted using an infinite impulse response. In this study, a median filter with a 100 Hz sampling frequency was applied. An RADWT (rational-dilation wavelet transform) was used to obtain the rational dilation factors from EEG signals at different scales with greater flexibility and accuracy. In contrast with conventional wavelet transformation, integer dilation factors are used in rational scaling. An RADWT is represented as a fraction (p/q). p and q represent integers that enhance the EEG signal. The filtered EEG signal enhances anxiety-induced seizure regions. Figure 3A depicts the filtered signals of EEG ES and non-ES signals. Band-pass and median filters were applied in the preprocessing stage. In Figure 3B (continued), RADWTs (rational-dilation wavelet transforms) are used to represent test signal frequency responses, execute wavelet representation, determine the distribution of signal energy, and reconstruct signals from individual side bands.

### 2.3. Data Augmentation of the BONN EEG Signals

Data augmentation [1,2,3,40] is used in machine and deep learning to increase the number of data samples. The increased quantity of signals in the database, derived through improved, synthetized versions of the EEG seizure signals, increases prediction accuracy. Random data augmentation was applied to BONN EEG epileptic seizure signals, resulting in new samples with the original label categorization. Augmentation strategies solve the problem of requiring a large quantity of data for training purposes. In [1], an extreme learning machine wavelet auto encoder method was used alongside augmented data and a wavelet activation function to enhance the sample variety through data augmentation. In this study, the random data augmentation of EEG signals allowed the mimicry of anxiety-induced seizure signals. Here, the ES signals are varied in terms of width, amplitude (A), and the combination of width and amplitude.

#### Random Data Augmentation

**Figure 4:**
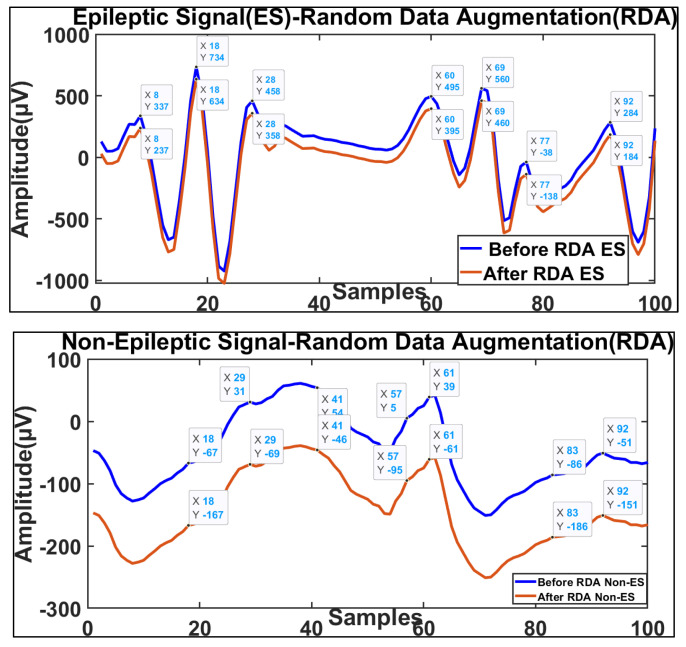
RDA based (anxiety seizure) EEG ES and non−ES.

In this study, random data augmentation was used to increase the diversity and size of the seizure signals in the EEG dataset. Data variation was achieved through random modifications. The diversity of the dataset was brought about by random data augmentation, which is a more effective technique in this regard. RDA creates EEG signals with varying angles, viewpoints, noise levels, and anxiety signals, mimicking anxiety-induced epileptic seizure signals through random changes. Figure 4 shows RDA-based (anxiety-induced seizure) EEG ES and non-ES signals. RDA alters or reduces the amplitude of the epileptic signals. The signals are iterated, and the amplitude of the epileptic signals is reduced. The amplitude of the EEG signals is reduced through RDA, a strategic method that enhances model performance, improves generalization, and maintains the signal characteristics for accurate anxiety detection. RDA creates a variety of EEG signals and improves the model accuracy through increasing the variety of features in the augmented signals. This diversity solves class imbalance problems in datasets and enhances model performance. The model learns from the balanced dataset, and overfitting is reduced through data augmentation, such as position and random data augmentation.

## 3. Results

The analysis was performed on an operating system with an Intel Core i3-1005G1, a CPU operating @ 1.20GHz, and 8 gigabytes (GBs) of RAM (Dell, China). MATLAB R2021a was utilized. The effectiveness of the classifier was determined.

### 3.1. Feature Extraction via Fuzzy Classification

**Figure 5:**
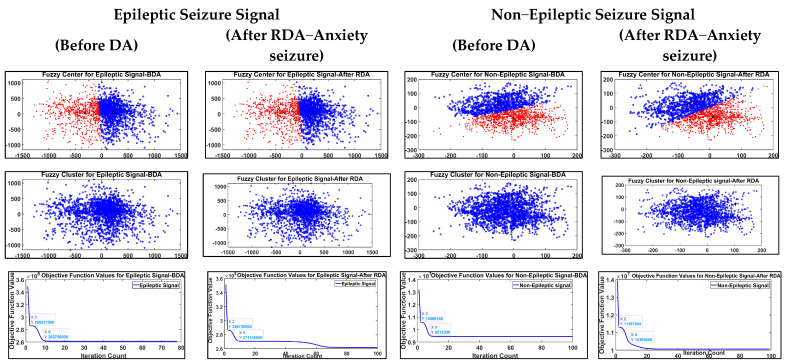
RDA signals applied with FCM for EEG ES and Non−Es signals feature extraction.

Fuzzy classifiers are used to extract features from RDA-augmented EEG signals. Fuzzy logic is used for feature extraction and extracts the pertinent features in EEG signals. A fuzzy classifier uses fuzzy logic principles, extracting features and selecting the pertinent features based on the features belonging to a class. Fuzzy logic enhances classification performance through the efficient management of imprecision and uncertainty in data. Fuzzy logic can be used to manipulate imprecise or uncertain data. Fuzzy classifiers are based on membership degrees, where a fuzzy set represents the uncertainty. The inference rule in fuzzy logic deals with ambiguous information. Fuzzy logic includes a set of mathematical operations. Fuzzy classifiers handle ambiguous and complex data and can be used to extract features. Fuzzy features are based on the characteristics of each class, and they are used to assess the relevant features. Higher-membership-degree features are selected for classification. The "if–then" rule is used for decision-making in fuzzy systems. For classification, each rule combines a number of attributes and membership functions. Fuzzy logic solves problems with inherent ambiguity and deals with imprecise or uncertain facts. Figure 5 depicts RDA signals processed with FCM for EEG ES and non-ES signal feature extraction. In Figure 5, the features were determined using FCM. For the EEG and random data-augmented signals (in which there is a reduction in the amplitude of the original epileptic EEG signal), the fuzzy cluster, fuzzy centre, and objective function are shown.

### 3.2. PSO-LSTM for Classification of EEG Signal

RDA increases efficacy, efficiency, and EEG signal quality. Particle swarm optimization is used for optimizing data or tasks involving data. PSO simulates the action of a swarm and obtains the best answer inside a certain issue domain. PSO is used in data optimization for feature selection, parameter tweaking, data clustering, and preprocessing. PSO optimizes data. In this approach, every outcome is called a particle (bird) [41] and described as a vector. The population (swarm), in this case, is made up of an arbitrary number of initial solutions. Every particle has a starting location and a velocity as it moves across the solution space and ultimately generates an optimal result. The initial steps of PSO are the determination of the initial position and velocity of each particle, followed by the upgrading of the variables for a predetermined number of generations, resulting in the best possible answer. 

Particle $\vec{d_{x}}$ is in ‘n’-dimensional space and represented as in Equation (1).

(1) $$\vec{d_{x}}=\left\{d_{x1},d_{x2},d_{x3},\;\ldots ,d_{xn}\right\}$$

x = 1, 2, 3, …, j, j represents particles number. Each particle are at different speed and represented as in Equation (2).

(2) $$\vec{s_{x}}=\left\{s_{x1},s_{x2},s_{x3},\;\ldots ,s_{xn}\right\}$$

Optimal solution is the ‘g_best_’ and every particle is with unique best position, denoted as ‘P_best_’ Particles move towards the best solution during iteration after changing their position and velocity in Equations (3) and (4).

(3) $$\vec{s_{x}}\left(t+1\right)=\lambda *\vec{s_{x}}\left(t\right)+c_{1}*r_{1}*\left(\vec{p_{x}}\left(t\right)-d_{x}\left(t\right)\right)+c_{2}*r_{2}*\left(\vec{g_{x}}\left(t\right)-\vec{d_{x}}\left(t\right)\right)$$

(4) $$\vec{d_{x}}\left(t+1\right)=\vec{d_{x}}\left(t\right)+\vec{v_{x}}\left(t+1\right)$$

Here, $\vec{s_{x}}\left(t+1\right)$ is the xth-velocity particle during iteration ‘t + 1’. λ stands for weight inertia, and $\vec{s_{x}}$ is the xth-velocity particle at iteration ‘*t*’. $\vec{p_{x}}\left(t\right)$ and $\vec{g_{x}}\left(t\right)$ represent the particle best and swarm global best, respectively, which are based on iteration ‘t’. $\vec{d_{x}}\left(t\right)$ and $\vec{d_{x}}\left(t+1\right)$ represent the past and present solutions. The cognition and social coefficient are represented by two positive real constants: c_1_ and c_2_. Random numbers between 0 and 1 are created for r_1_ and r_2_. A recurrent neural network, LSTM [30,31], solves the vanishing gradient problem, making it possible for the network to identify and monitor the long-term dependencies in a collection of data. RNNs are less complex in structure than LSTM networks and require unique memory cells, which hold information for long periods of time. The memory cells are connected through a gate system, which regulates the flow of information. The three main gates of an LSTM are input, forget, and output. These gates control the information that enters and leaves memory cells, as well as whether data are retained or deleted from those cells. An input gate is added to the memory cells and refreshes their values. It considers both the input at a given moment and that in previous hidden states. The forget gate selects data from the memory cell to discard. It outputs a forget factor for every memory cell by considering both the past concealed state and the current input. The quantity of data taken from memory cells is controlled through output gates. The considerations here include the current input, historical biases concealed in the state information, and outputs in a hidden state at the current time step. To update and store information in memory cells, LSTM combines multiplicative and additive interactions and allows for the long-term retention or forgetting of specific knowledge. The technique used to train LSTM is backpropagation, which updates a network’s weights and biases.

### 3.3. Hyperparameter Tuning Using PSO in LSTM

Hyperparameter tuning was performed using an optimization algorithm. Tuning techniques improve performance, accelerate the process, and determine the ideal hyperparameter values. There are numerous hyperparameter optimization techniques, such as particle swarm optimization, grid search, and Bayesian optimization. The selection of the ideal value for a layer, such as the learning rate, neurons, activation functions, dropout rate, or batch size optimizer, is called hyperparameter tuning. The framework for PSO tuning in LSTM and parrot optimization is depicted in Table 2.

### 3.4. Proposed Methods

#### 3.4.1. FCM-PS-LSTM

**Figure 6:**
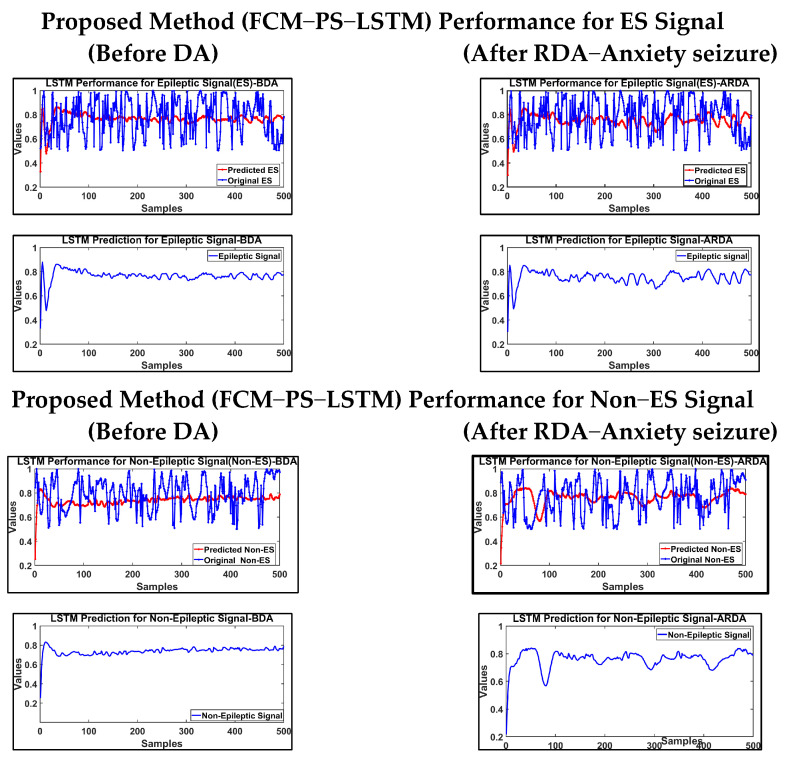
Proposed FCM−PS−LSTM method in classification of EEG ES and Non−ES signal.

The FCM classifier was used to extract the statistical properties of epileptic and non-epileptic EEG signals. A PSO hyperparameter-tuned LSTM classifier process with fuzzy logic was used to extract the features and classify the epileptic and non−epileptic EEG signals. This technique solves the problem of overlapping clusters, as PSO finds optimal solutions in large search spaces. PSO performs better than other algorithms in this regard due to its adaptability and convergence properties, enabling it to find the optimal solutions through dynamically adjusting the search parameters. LSTM is a sequential data processing technique and maintains long-term dependencies in data sequences, making it appropriate for EEG signals. LSTM predicts values in PSO algorithms, reduces the fitness value, and increases optimization efficiency. In this study, 70% of the data were used for training, and 30% of the data were used for testing. Here, 10-fold cross validation was used for analysing the data. The performance of FCM−PS−LSTM is shown in Figure 6.

#### 3.4.2. PS−LSTM

**Figure 7:**
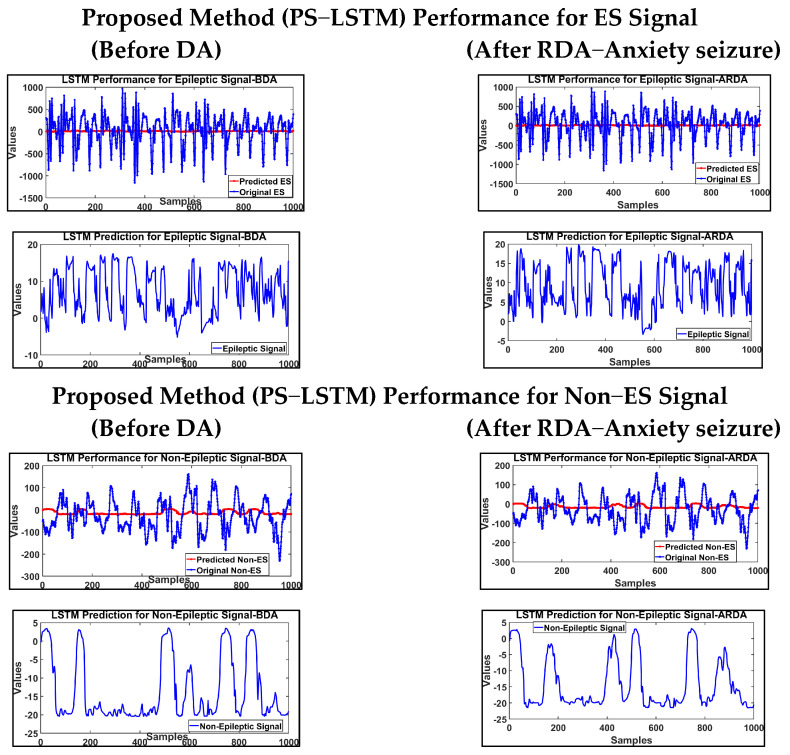
Proposed PS−LSTM method in classification of EEG ES and Non−ES signal.

The LSTM classifier classified non-epileptic and epileptic EEG signals using PSO hyperparameter tuning. The performance of the proposed (PS−LSTM) method for ES and non−ES signals is depicted in Figure 7.

#### 3.4.3. Parrot Optimization-LSTM

**Figure 8:**
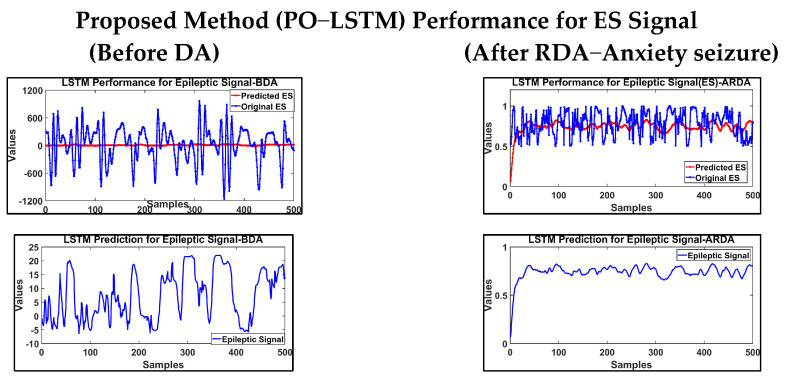

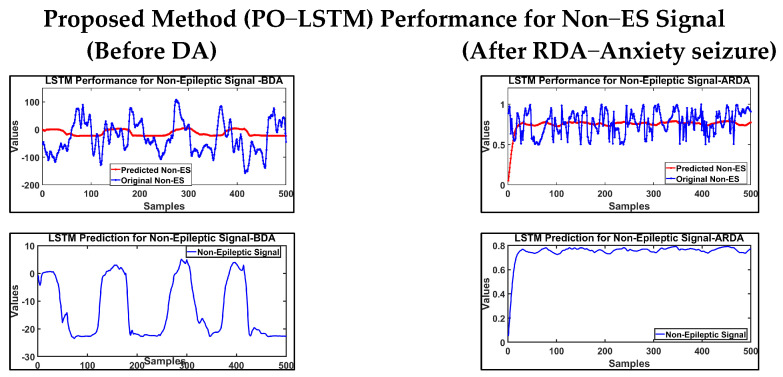
Proposed PO−LSTM method in classification of EEG ES and Non−ES signal.

The observed behaviour of a trained parrot, Pyrrhura Molinae, served as the model for the parrot optimizer (PO) optimization technique [42]. This technique addresses the optimization issues present in a variety of domains. POs are used in deep learning and to optimize neural network hyperparameters. The parrot optimizer was used to optimize the LSTM hyperparameters. The dataset was split into five folds for this process, and the network was iteratively retrained and validated using cross-validation and its performance was estimated. The cumulative cross-validation data provided by parrot optimization yielded the optimal hyperparameters, and these were used to train the network and subsequently evaluate the test data. The performance of the proposed PO−LSTM method for ES and non−ES EEG signals is depicted in Figure 8.

##### Mathematical Model of PO

Initialization of population

$$Y_{i}^{0}=lb+\mathrm{rand}\left(0,1\right)*\left(ub-lb\right)$$

Here, lb denotes the lower band and ub denotes the upper band, *rand*(0,1) denotes a random number in the range [0, 1], and $X_{i}$ denotes the position of the *i*th Pyrrhura Molinae in the initial phase.

Foraging behaviour

$$Y_{i}^{t+1}=(Y_{i}^{t}-Y_{best})\;.levy\left(dim\right)+rand\left(0,1\right)\;.{\left(1-\frac{t}{Max_{iter}}\right)}^{\frac{2t}{Max_{iter}}}.Y_{mean}^{t}$$

Staying Behavior

$$Y_{i}^{t+1}=Y_{i}^{t}+Y_{best}\;.levy\left(dim\right)+rand\left(0,1\right)\;.ones\left(1,dim\right)$$

Communicating Behavior

$$Y_{i}^{t+1}=\{\begin{array}{c} 0.2\;.rand\left(0,1\right)\;.\left(1-\frac{t}{Max_{iter}}\right)\left(Y_{i}^{t}-Y_{mean}^{t}\right),\;P\leq 0.5\\ 0.2\;.rand\left(0,1\right)\;.\;e^{\frac{-t}{rand\left(0,1\right).Max_{iter}}}\;\;,\;P>0.5 \end{array}$$

Fear of strangers’ behavior

$$Y_{i}^{t+1}=Y_{i}^{t}+rand\left(0,1\right)\;.\cos\left(0.5\pi .\frac{t}{Max_{iter}}\right)\;.\;\left(Y_{best}-Y_{i}^{t}\right)\;\;-\cos\left(rand\left(0,1\right).\pi \right).{\left(\frac{t}{Max_{iter}}\right)}^{\frac{2}{Max_{iter}}}\;.\left(Y_{i}^{t}-Y_{best}\right)$$

### 3.5. Performance Metrics

**Figure 9:**
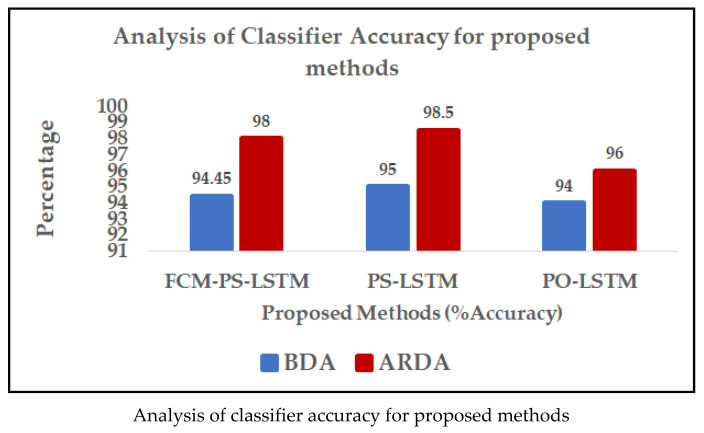

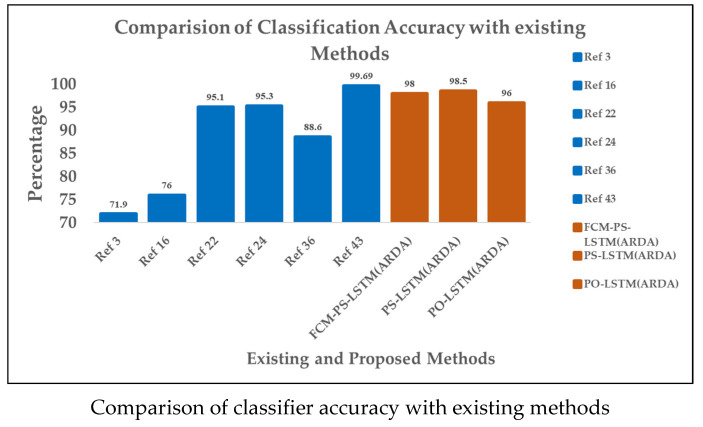
Comparison of Classifier Accuracy with Existing and Proposed Methods.

Evaluation metrics were used to determine the proposed method’s efficacy in classifying anxiety-based EEG ES signals. Table 3 depicts the results for the classifier accuracy analysis for the proposed method. Increased perturbations lead to decreased accuracy in RDA-generated anxiety-related effect sizes, but employing exact data-preprocessing, feature selection, and validation algorithms mitigates the effects of these issues and enhances the reliability of the results. The various classifiers’ performance metrics for the signals before data augmentation (BDA) and after random data augmentation (ARDA) in regard to EEG epileptic signals are compared in Table 4. In this paper, the results of the proposed methods, (i) FCM-PS-LSTM, (ii) PS-LSTM, and (iii) PO-LSTM, are compared using metrics such as accuracy, specificity, sensitivity, F1-Score, MCC (Matthew’s correlation coefficient), CSI, kappa, precision, and FM index. A comparison of the classification accuracy of the existing methods with that of the proposed methods is shown in Figure 9. Table 5 depicts a comparison of the classification accuracy between the existing and proposed approaches for EEG epileptic seizure signals.

## 4. Discussion

Epilepsy is a condition which arises when aberrant signals are transmitted by a number of nerve cells, resulting in seizures. Brain electrical activity is measured using electroencephalograms (EEGs). Epileptic seizures caused by anxiety are reflected in EEG signals through changes in a signal’s size and form. The intent of this EEG-based research is to investigate more precise and effective classification techniques. We propose techniques to identify anxiety-based EEG signals corresponding to epileptic seizures and non-epileptic seizures. For analysis, data augmentation has been applied to emotional EEG signals [1,3] and sleep-related EEG signals [15]. Epileptic seizure signals from the Bonn dataset were data-augmented for anxiety-based EEG signals. Statistical attributes [22] such as Hjorth activity, Kurtosis, mean, standard deviation, Shannon entropy, skewness, and variance were extracted through a fuzzy classifier. The data-augmented anxiety seizure signals were processed with three proposed algorithms: (i) fuzzy C-means particle swarm optimization–long short-term memory, (ii) particle swarm optimization–long short-term memory, and (iii) parrot optimization–LSTM. In [19], PSO for optimization and BPNN were used for the classification of seizures. In [43], the author conducted an evaluation of the preprocessing and feature extraction of seizure predictions using EEG time series data. The authors of [44] employed an NN model based on MDSN for seizure detection and achieved an accuracy of 99.69%. In this study, hyperparameter-tuned PSO and PO were used for an LSTM classifier. Among the above-stated proposed methods, PS-LSTM achieved 98.5% for ARDA and 95% for BDA. The classifier performance was compared with that of LR, GNB, and MLR. In the future, EEG epileptic seizure classifications for tonic–clonic, myoclonic, febrile, and atonic seizures need to be obtained through the data augmentation of signals, and an analysis of the effect of gender peculiarities on classification efficacy using the proposed approach must be conducted.

## 5. Conclusions

In this study, we used RDA, fuzzy-based feature extraction, and the hyperparameter tuning of PSO. PO-tuned LSTM was used for the diagnosis of anxiety-based EEG ES and non-ES signals. Statistics from fuzzy bases such as Hjorth activity, Kurtosis, mean, standard deviation, Shannon entropy, skewness, and variance were extracted and analysed. In this paper, we propose the following classification methods: (i) FCM-PS-LSTM, (ii) PS-LSTM, and (iii) PO-LSTM. These methods achieved accuracies of about (i) 98%, (ii) 98.5, and (iii) 96%, respectively. 

## Figures and Tables

**Table 1:** The description of the BONN EEG Epileptic Seizure dataset.

Sets	Subjects				
	Patient Phase	Electrode Kind/Location	No. of Study	Number of Data	Length of Sections
Set-A	Eye open	Surface (subject skin)	5	100	4097
Set-B	Eye close	Surface (subject skin)	5	100	4097
Set-C	Seizure free (Non-Epileptic)	Intracranial (skull)	5	100	4097
Set-D	Seizure Free	Intracranial (skull)	5	100	4097
Set-E	Seizure Activity (Epileptic)	Intracranial (skull)	5	100	4097

**Table 2:** Framework for PSO tuning in LSTM and Parrot Optimization.

Description	Values Used	Reference	
Particle Swarm Optimization (PSO)			
Size of swarm	9	-	-
Maximum value of repetitions	100	-	-
C_1_ (Cognition Coefficient), C_2_ (Social Coefficient)	C_1_ = C_2_ = 2	1.4962 [21]	0.9 [41]
**LSTM**			
Gradient Threshold	0.01	-	-
Learning rate	0.0001	0.001 [29]	0.005 [30]
No. of hidden units	100	64 [29]	-
Input layer	Sequence layer	-	-
Activation function	tanh(state), Sigmoid(gate)	SoftMax [29]	-
Output layer	Regression layer	-	-
Drop out	0.5	-	-
**Parrot Optimization**			
Maximum Iteration	1000	[42]	
Lower Bound (lb)	−100		
Upper Bound (ub)	100		
Number of search agents	30		
Dimension of search space	30		

**Table 3:** Analysis of classifier accuracy for stated methods.

Methods	FCM-PS-LSTM	PS-LSTM	PO-LSTM
	Accuracy (%)		
Before Data Augmentation (BDA)	**94.45**	**95**	**94**
After Random Data Augmentation (ARDA)	**98**	**98.5**	**96**

**Table 4:** Comparison of the classifier’s outcomes for anxiety-based ES using ARDA and BDA.

Methods	BDA						ARDA					
Classification Effectiveness	LR	GNB	MLR	FCM-PS-LSTM	PS-LSTM	PO-LSTM	LR	GNB	MLR	FCM-PS-LSTM	PS-LSTM	PO-LSTM
**Accuracy**	93.33	68.3	91.66	**94.45**	**95**	**94**	96	85.5	97.5	**98**	**98.5**	**96**
**Sensitivity**	93.7	82.35	90.9	94.94	94.11	95	97	84.84	97	97	98	97
**Specificity**	92.8	62.79	92.59	94	95.91	93	95	86.13	97.97	98.9	98	95
**Precision**	93.75	46.66	93.75	94	96	93.13	95.09	85.7	98	98.9	98	95.09
**F1-Score**	93.75	59.57	92.3	94.47	95.04	94.05	96.03	85.27	97.51	98	98.4	96.03
**MCC**	86.6	40.6	83.2	89.0	90.0	88.01	66.4	54.0	68.3	68.9	69.1	92.01
**Kappa**	86.6	36.6	83.2	89	90	88	92.1	72	95.03	96	97	92
**CSI**	88.2	42.4	85.7	89.5	90.5	88.7	92.3	74.3	95.1	96.1	97	92.3
**FM Index**	93.72	61.98	92.3	94.46	95.04	94.06	96.04	85.26	97.49	98.44	98	96.04

LR—logistic regression; GNB—Gaussian naïve Bayes; MLP—multiple linear regression; Proposed Methods: (i) FCM-PS-LSTM, (ii) PS-LSTM, and (iii) PO-LSTM; BDA—before data augmentation. ARDA—after random data augmentation.

**Table 5:** Comparison of classifier accuracy with latest methods for EEG Epileptic seizures signal.

Title	Purpose	Database	Strategy			Evaluation Metrics								
			PP	Features	Classifier									
EEG Feature Extraction and Data Augmentation in Emotion Recognition [3]	Detection of Arousal and valence	DEAP dataset (Emotional)	CWGAN for data augmentation	Average PSD, Zero Crossing rate, Mean, variance for traits	SVM, DNN	Accuracy 71.9%								
Staging Study of Single-Channel Sleep EEG signals Based on Data Augmentation [15]	Detection of sleep period(wake,N1,N2,N3,REM)	SC subset of Sleep-EDF Database	RDB-DCGAN data augmentation model. Wavelet time frequency transform		CNN	Accuracy 76%								
Classification of Epileptic EEG Signals Using PSO-Based Artificial Neural Network and Tunable-Q Wavelet Transform [21]	Categorization of Epileptic EEG signals(Normal/Focal/Generalized)	KIT TUH	TQWT	Non-linear attributes such as log energy entropy, Shannon entropy and Stein’s unbiased risk estimate entropy. PSO	ANN	Accuracy: (i) normal–focal (95.1%), (ii) normal–generalised (97.4%), (iii) normal–focal + generalized (96.2%), and (iv) normal–focal generalized (88.8%) for TUH								
Epileptic Seizure Prediction Based on Hybrid Seek Optimization Tuned Ensemble Classifier Using EEG signals [22]	Prediction of Epileptic seizure	Siena database CHB-MIT	BPF	Statistical, Wavelet and Entropy-based attributes	DT, RF & AdaBoost classifier	Siena			CHB-MIT					
						Accuracy 95.3% Sensitivity 93.17% Specificity90.06%			Accuracy 96.6% Sensitiivty 94.67% Specificity 91.36%					
Prediction of Seizure in the EEG Signal with Time Aware Recurrent Neural Network [33]	Prediction of EEG seizure (Inter-ictal)	CHB-MIT BONN VIRGO EEG	Time Aware CNN and Recurrent Neural Network (TA-CNN-RNN) Model		LSTM	CHB-MIT			BONN			VIRGO EEG		
						Accuracy 89% Precision 88.3% Recall 91.3% F-measure 89.8%			Accuracy 88.6% Precision 87.7% Recall 90.9% F-measure 89.2%			Accuracy 88.7% Precision89.4% Recall 92.4% F-measure 90.7%		
Training Datasets for Epilepsy Analysis: Preprocessing and Feature Extraction from Electroencephalography Time Series [43]	Seizure Prediction	Freiburg Databas.	Sliding window Techniques and multiple features are extracted TrBtool used		NA	NA								
Detection Method of Epileptic Seizures Using a Neural Network Model Based on Multimodal Dual-Stream Networks [44]	Seizure detection	Bonn EEG dataset New Delhi Dataset	Short time Fourier transform	signal differential attributes, frequency domain amplitude spectrum and phase spectrum methods	Multimodal dual stream networks	99.69%Accuracy, 99.44%Precision, 1%Recall, 99.72%F1-score for Bonn EEG dataset								
**Proposed Methods**	EEG Epileptic seizure (anxiety based)	BONN EEG dataset	BPF, Median and RADWT	Statistical	LSTM **(i) PFCM-PS-LSTM** **(ii) PS-LSTM** **(iii) PO-LSTM**	**ARDA(%)**								
						**Accuracy**	**Sensitivity**	**Speficity**	**Precision**	**F1-score**	**MCC**	**Kappa**	**CSI**	**FM Index**
						98	97	98.9	98.9	98	68.9	96	96.1	98.44
						98.5	98	98	98	98.4	69.1	97	97	98
						96	97	95	95.09	96.03	92.01	92	92.3	96.04

## Data Availability

The data that support the findings of this study are openly available in http://epileptologie-bonn.de/cms/front_content.Php?idcat=193&lang=3, https://www.ukbonn.de/epileptologie/arbeitsgruppen/ag-lehnertz-neurophysik/downloads/ (Accessed on 10 August 2023).
